# [Expression of Concern] Luteolin suppresses TNF-α-induced inflammatory injury and senescence of nucleus pulposus cells via the Sirt6/NF-κB pathway

**DOI:** 10.3892/etm.2026.13315

**Published:** 2026-09-29

**Authors:** Tian Xie, Jun Yuan, Ling Mei, Ping Li, Ruijie Pan

Exp Ther Med 24:469, 2022; DOI: 10.3892/etm.2022.11396

Following the publication of this paper, it was drawn to the Editor's attention by a concerned reader that the one of the antibodies the authors used in their study was apparently selected inappropriately: regarding the western blot experiments shown in Figs. 3C and 6C, the antibody (cat. no. ab51243) that the authors reported to have used for the apoptosis-associated protein p16-INK4a was in fact specific for the protein known as p16 ARC (encoded by the ARPC5 gene), not as the authors had intended.

The authors have been contacted by the Editorial Office to offer an explanation for the apparently incorrect selection of an antibody in this paper, and we are awaiting their response. Owing to the fact that the Editorial Office has been made aware of a potential issue surrounding the scientific integrity of this paper, we are issuing an Expression of Concern to notify readers of this potential problem while the Editorial Office continues to investigate this matter further.

